# BioID identifies proteins involved in the cell biology of caveolae

**DOI:** 10.1371/journal.pone.0209856

**Published:** 2018-12-27

**Authors:** C. Mendoza-Topaz, I. Yeow, K. Riento, B. J. Nichols

**Affiliations:** MRC Laboratory of Molecular Biology, Cambridge, United Kingdom; Institut Curie, FRANCE

## Abstract

The mechanisms controlling the abundance and sub-cellular distribution of caveolae are not well described. A first step towards determining such mechanisms would be identification of relevant proteins that interact with known components of caveolae. Here, we applied proximity biotinylation (BioID) to identify a list of proteins that may interact with the caveolar protein cavin1. Screening of these candidates using siRNA to reduce their expression revealed that one of them, CSDE1, regulates the levels of mRNAs and protein expression for multiple components of caveolae. A second candidate, CD2AP, co-precipitated with cavin1. Caveolar proteins were observed in characteristic and previously un-described linear arrays adjacent to cell-cell junctions in both MDCK cells, and in HeLa cells overexpressing an active form of the small GTPase Rac1. CD2AP was required for the recruitment of caveolar proteins to these linear arrays. We conclude that BioID will be useful in identification of new proteins involved in the cell biology of caveolae, and that interaction between CD2AP and cavin1 may have an important role in regulating the sub-cellular distribution of caveolae.

## Introduction

Caveolae are flask-shaped invaginations of the plasma membrane found in many vertebrate cell types. They are especially abundant in endothelial cells, adipocytes and muscle cells [1–3]. A range of functions have been attributed to caveolae, including roles in endocytosis, organisation of plasma membrane signalling molecules, regulation of membrane lipid composition, and protection of cells from mechanical stress forces within the membrane [1, 3–9]. The molecular basis of all of these potential functions is under active investigation.

The protein complexes required for assembly of caveolae are increasingly well characterised. Fundamental components include caveolins—membrane proteins embedded in the cytosolic face of the membrane, and cavins—trimeric coiled-coil-forming proteins that are recruited from the cytoplasm to caveolae in the presence of caveolins [10, 11]. Both caveolin1 and cavin1 are essential for formation of caveolae [9, 12, 13]. As well as being present at caveolae, cavin1 has additional functions within the nucleus, where it regulates ribosomal RNA synthesis [14–17]. Caveolins and cavins can, in the presence of chemical cross-linkers, be purified as a single caveolar coat complex that has the size and shape of the membrane bulb of caveolae [18, 19]. There are separate complexes at the neck of caveolae, made up from EHD (Eps15 Homology Domain) proteins and potentially members of the pacsin and dynamin protein families [20–24]. EHD proteins are important for the propensity of caveolae to form interlinked clusters or arrays, and may be important for reversible changes in caveolar morphology [20]. Importantly, the caveolar coat complex has been highly purified after chemical cross-linking, and analysed by mass spectrometry [18, 19]. There are no further abundant components of the complex other that cavin and caveolin proteins.

Given the above, it is possible that the ‘parts list’ of key proteins required for the assembly and structural integrity of caveolae is now complete [10]. There are, however, many aspects of the cell biology of caveolae that are incompletely understood and are likely to involve still unknown protein-protein interactions. If caveolae are involved in signal transduction processes then mechanisms are likely to exist to relay signals from caveolae to the cytoplasm [8, 25]. The distribution of caveolae in the cell is clearly non-stochastic, caveolae migrating to the back of motile cells and aligning parallel to actin stress fibres, and these distributions are likely to be mediated by interactions between caveolae and the elements of the cortical cytoskeleton [2]. Caveolae apparently bud from the plasma membrane and move through the cytoplasm on linear microtubule tracks, implying recruitment of motor proteins and related factors [26, 27]. It is therefore likely that understanding the nature of the protein-protein interactions undergone by components of caveolae will provide considerable insights.

There is a large literature reporting interactions between caveolin1 and other proteins. The physiological significance, however, of some of the reported interactions has been questioned by more recent experiments showing that a putative caveolin binding domain found in several interaction partners may not be structurally suitable to interact with caveolin1 [28]. Caveolin1 itself forms oligomers within the plasma membrane that are highly resistant to extraction with non-ionic detergents, making co-immunoprecipitation experiments potentially difficult to conduct and interpret [10, 29, 30]. Moreover, the types of interaction governing, for example, sub-cellular localisation of caveolae could be transient, low affinity and regulated both temporally and spatially within the cell. These issues prompted us to take an alternative approach to identifying interaction partners for cavin1, one of the core components of caveolae.

We employed proximity biotinylation to identify potential interaction partners for cavin1 [31, 32]. We successfully identified known components of caveolae, and 13 additional candidates. Screening of the additional candidates using siRNA knockdowns and co-immunoprecipitation experiments revealed CSDE1 and CD2AP as especially likely to interact functionally with caveolar components. We found that CSDE1 controls both protein and mRNA levels for multiple components of caveolae, through an unknown mechanism. We found CD2AP to be specifically co-precipitated with cavin1 and caveolin1, and hence this candidate was selected for further investigation. We present data to show that CD2AP controls the recruitment of caveolae to membrane zones adjacent to cell-cell junctions, providing a mechanistic link between caveolae and the cortical cytoskeleton. We conclude that proximity biotinylation provides a useful approach with detecting interactions with caveolar components that may be transient, of low affinity or otherwise refractory to conventional biochemical approaches.

## Results

### BirA* fusions for identification of proteins that interact with cavin1

**Fig 1:**
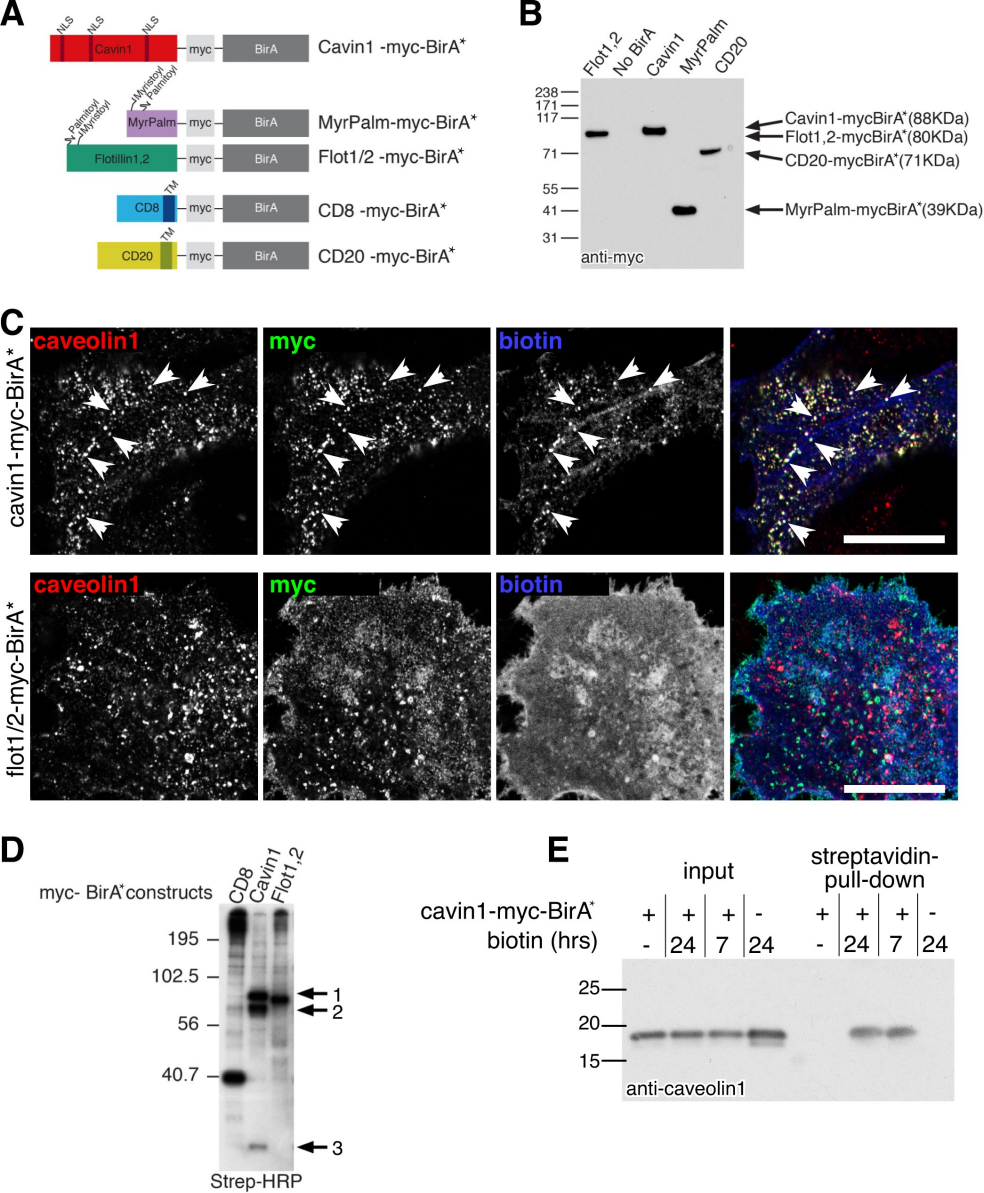
Expression of cavin1-myc-BirA* as a tool to label caveolar proteins. **A**. Schematic representation of constructs used in this study. **B**. Expression of constructs used in this study, analysed by Western blotting with anti-myc antibodies. **C**. Distribution of biotin in transfected cells, compared with caveolae labelled with antibodies against caveolin1. Anti-myc antibodies reveal the location of the indicated BirA* construct. Streptavidin reveals the location of biotinylated proteins. Arrowheads highlight examples of streptavidin-stained caveolae. Bar is 20 microns. Single confocal sections acquired with 63x objective. **D**. Blot of biotinylated proteins labelled with streptavidin-HRP. Cells were transfected with the myc-BirA* construct indicated at the top of each lane on the blot, and incubated with exogenous biotin before solubilisation. The band labelled 1 in the cavin1-myc-BirA* lane is the correct size to be cavin1-myc-BirA*, 2 is the correct size to be endogenous cavin1, and 3 is the correct size to be caveolin1. **E**. Western blot labelled with caveolin1 antibody. Cells were transfected with cavin1-myc-BirA* and incubated with exogenous biotin for the times shown, before solubilisation and precipitation of biotinylated proteins with immobilised streptavidin.

Proximity biotinylation is potentially a powerful technique to detect transient, low affinity or highly regulated interactions [31, 32]. Extensive controls are, however, required to facilitate interpretation. We produced a construct for expression of cavin1 fused to the promiscuous biotin ligase BirA*, and a range of constructs to serve as negative controls: 1. A version of BirA* with myristoylation and palmitoylation sites that will effectively target the resultant fusion protein to the plasma membrane, 2. BirA* fused to both flotillin1 and to flotillin2, plasma membrane proteins that reside in microdomains that are distinct from caveolae [33], 3. BirA* fused to CD8, a plasma membrane protein with a trans-membrane domain, BirA* being expressed on the cytoplasmic side of the membrane. 4. BirA* fused to CD20, a second trans-membrane-domain plasma membrane protein (Fig 1A). A copy of the myc epitope was included in all of the constructs (Fig 1A), and transient transfection of HeLa cells resulted in similar expression levels (Fig 1B).

Initially, we carried out experiments to ascertain whether cavin1-myc-BirA* is recruited to caveolae. Using the myc epitope included in the construct, we applied indirect immunofluorescence to show that cavin1-BirA* is recruited to caveolin1-positive caveolae just as endogenous cavin1 (Fig 1C). Furthermore, incubation with biotin followed by labelling with fluorescent streptavidin confirmed that caveolin1- and cavin1-positive caveolae are the predominant sites of biotinylation within cells expressing the cavin1-myc-BirA* construct (Fig 1C). Comparison with the pattern of biotinylation produced by the negative control constructs, for example flotillin1/flotillin2-myc-BirA*, revealed that the negative controls produced a different pattern of biotinylation, with more uniform staining of the plasma membrane (Fig 1C).

Labelling of the total population of biotinylated proteins on blots with streptavidin-HRP, after incubation of the transfected HeLa cells with biotin, showed that different sets of proteins are biotinylated by the cavin1-myc-BirA* construct and by the negative controls (Fig 1D). Moreover, cavin1-myc-BirA* specifically biotinylated prominent bands with the correct size to be caveolin1 and endogenous cavin1 (Fig 1D). Precipitation of all biotinylated proteins with immobilised streptavidin was used to confirm that caveolin1 is specifically biotinylated in the presence of cavin1-myc-BirA*, as Western blotting of the eluted precipitate with anti-caveolin1 antibodies revealed caveolin1 to be present only when both exogenous biotin and cavin1-myc-BirA* were added (Fig 1E). These pilot experiments show, therefore, that the cavin1-myc-BirA* construct is recruited to caveolae and biotinylates a specific set of proteins present at caveolae including caveolin1.

**Fig 2:**
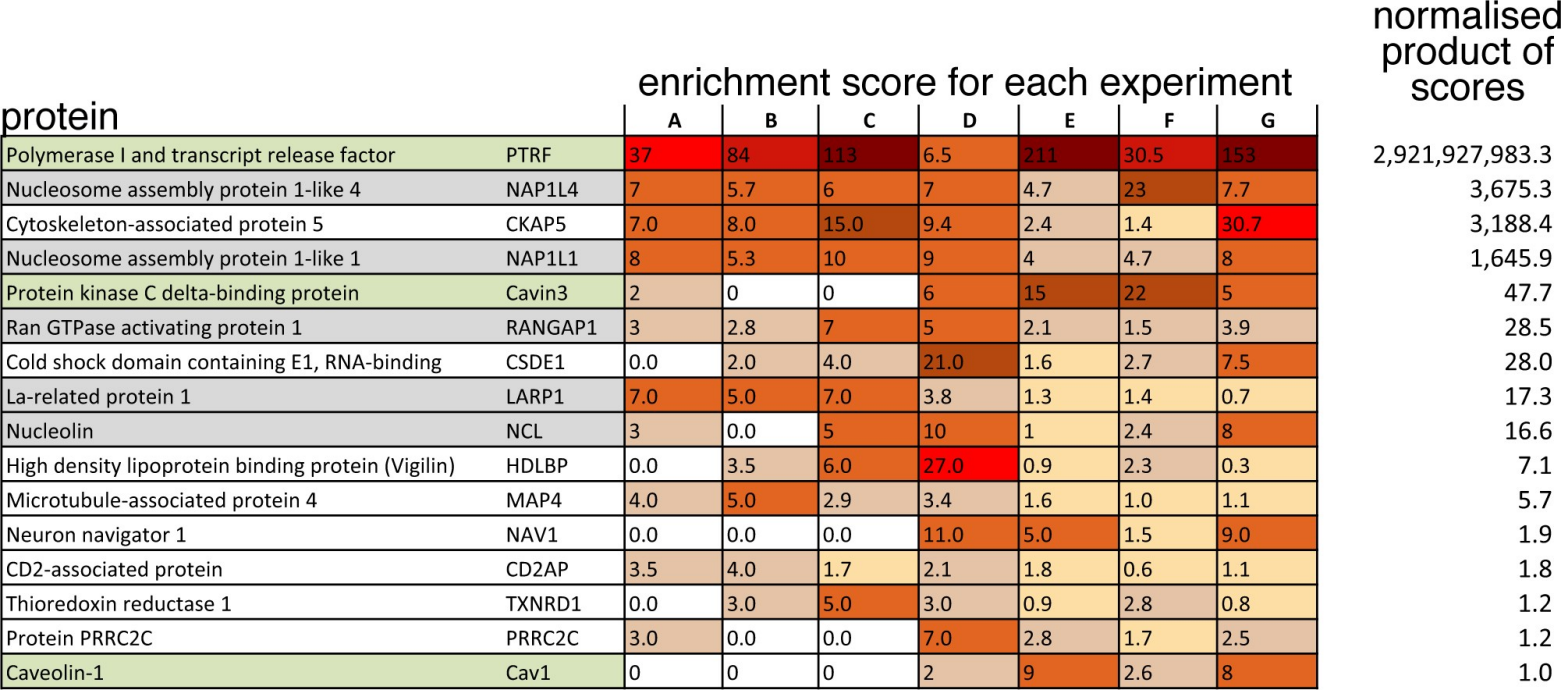
Candidate cavin1-interacting proteins identified by BioID. To show all proteins from 7 pooled BioID experiments with a normalised product of enrichment scores greater than that of caveolin1 –the known caveolar protein to which the scores were normalised. A score of zero means that the specified protein was not detected in that experiment. Protein names are colour coded: green–known caveolar component, grey–nuclear protein. To aid visualisation, enrichment scores are shaded with a higher score having stronger shading.

We carried out 7 separate experiments in which HeLa cells expressing cavin1-myc-BirA* and cells expressing negative control constructs were incubated with exogenous biotin for 16–20 hours before lysis and isolation of all biotinylated proteins using a streptavidin column, and identification of these proteins using mass spectrometry. Mass spectrometry data from all of the experiments is contained in S1 File. Pilot experiments had established 16–20 hours incubation with biotin as being sufficient to result in optimal biotinylation of a relatively small number of distinct bands, as in Fig 1D. In each individual experiment a score for enrichment of each identified protein in the cavin-1-myc-BirA* samples over the parallel negative controls was generated, and an overall enrichment score from all 7 experiments was calculated using the product of the enrichment scores for each experiment normalised so that the known caveolar component caveolin1 was set to 1. This method of aggregating the scores from individual experiments was selected as it favours candidates that were consistently found in all experiments over those with a high enrichment score in only a small subset of the experiments. Proteins with an overall enrichment score greater than that of caveolin1 are listed in Fig 2, and were selected for further analysis.

Fig 2 represents a set of potential interaction partners for cavin1. It contains cavin and caveolin proteins known to be expressed in the HeLa cells used for these experiments (green shading). It contains a number of proteins present in the nucleus, the function of which may be related to the nuclear function of cavin1 in regulating ribosomal RNA synthesis (grey shading) [14]. Encouragingly, it contains one further protein, thioredoxin reductase 1, that has previously been shown to co-localise with components of caveolae [34].

### CSDE1 regulates the levels of mRNAs for components of caveolae

**Fig 3:**
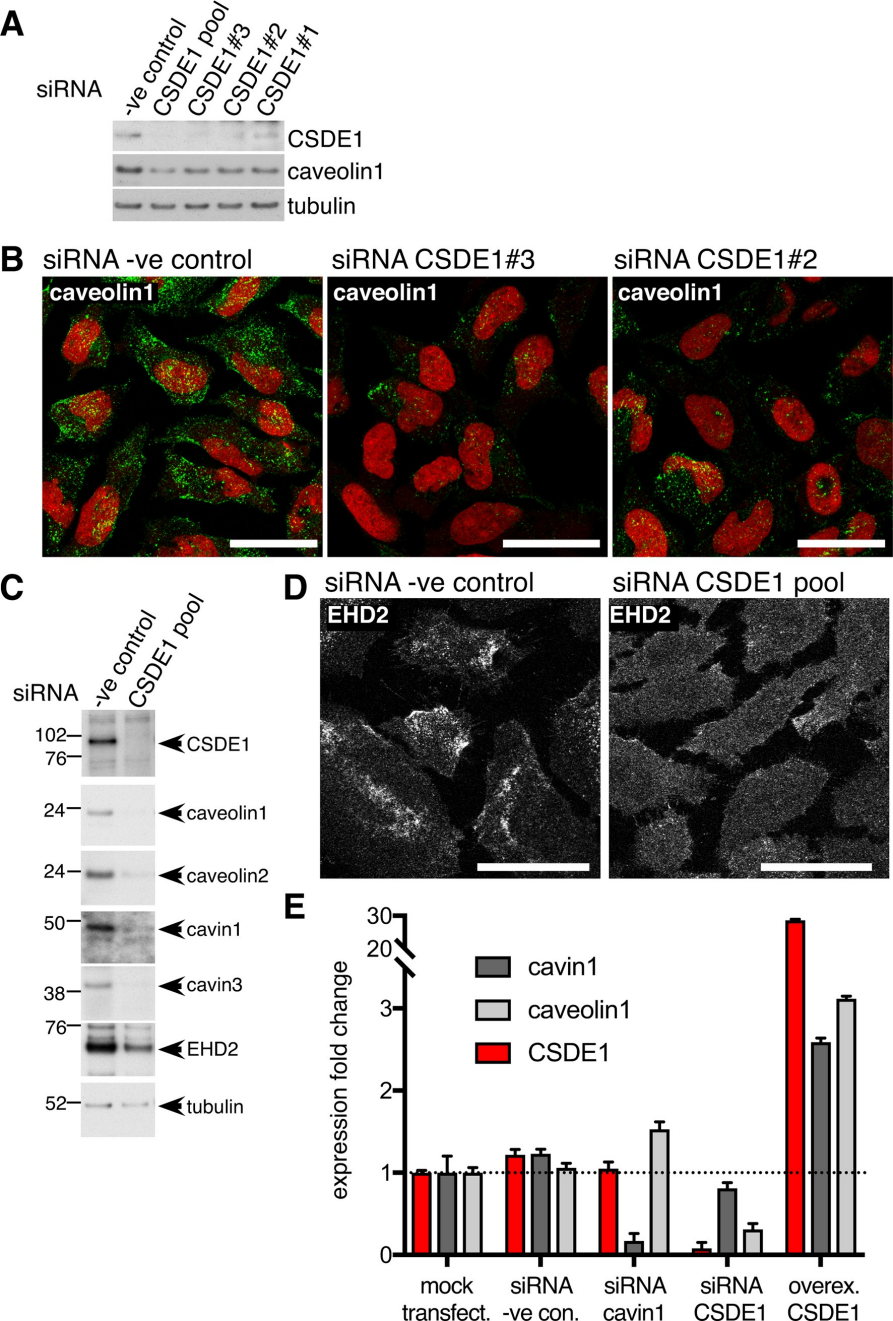
CSDE1 controls expression of components of caveolae. **A**. SiRNAs against CSDE1 reduce caveolin1 levels, as judged by Western blotting with the antibodies shown. Three different single siRNA species were used, as well as a pooled population. **B**. SiRNAs against CSDE1 reduce caveolin1 levels, as judged by indirect immunofluorescence. Two different single siRNA species were used. Cell nucleii are stained with propidium iodide. Bars 20 microns. Maximum intensity projections of multiple confocal sections acquired at 1 micron intervals, with 63x objective. **C**. SiRNAs against CSDE1 reduce levels of multiple caveolar components, as judged by Western blotting with the antibodies shown. A pooled population of siRNAs was used. **D**. SiRNAs against CSDE1 cause delocalisation of EHD2, consistent with loss of recruitment to caveolae. Bars 20 microns. Maximum intensity projections of multiple confocal sections acquired at 1 micron intervals, with 63x objective. **E**. Quantitative PCR to measure changes in cavin1, caveolin1 and CSDE1 mRNA levels relative to mock-transfected cells. Cells were transfected with the pooled siRNAs shown, or with plasmid for transient over-expression of CSDE1. Bars are SD, N = 3. The experiment was repeated twice with equivalent results.

We employed siRNA to deplete expression of selected candidate interaction partners for cavin1, and then Western blotting to ask whether expression of known caveolar components was altered (S1 Fig). Of the candidates tested, only siRNAs to CSDE1 produced a clear decrease in caveolin1 expression as revealed by both Western blotting and indirect immunofluorescence, using multiple independent siRNAs (Fig 3A, Fig 3B, S1 Fig). CSDE1 (Cold Shock Domain-containing E1), also called Unr (Upstream of N-ras), is an RNA-binding protein thought to control gene expression by post-translational mechanisms [35–37]. To confirm that CSDE1 is required for correct expression of caveolar proteins we carried out Western blotting to assess levels of caveolin1, caveolin2, cavin1, cavin3 and EHD2 in cells transfected with a combination of CSDE1 siRNAs that produces highly efficient reduction in CSDE1 expression (Fig 3C). Expression of all 5 caveolar components was clearly reduced. As an additional test of whether caveolae are perturbed by CSDE1 siRNAs, we stained siRNA-treated cells with antibodies to the caveolar protein EHD2 (Fig 3D). The siRNA-treated cells displayed a marked change in EHD2 distribution, consistent with the loss of the punctate, caveolin1-positive structures to which EHD2 is normally recruited (Fig 3D). Quantitative PCR revealed that there is a marked decrease in caveolin1 mRNA levels in CSDE1-siRNA-treated cells, in contrast to the effect of cavin1 siRNAs which increase caveolin1 mRNA levels (Fig 3E). This suggests that CSDE1 has an important role in regulating mRNA levels of caveolar proteins. In agreement with this, when CSDE1 is overexpressed there is a pronounced increase in the levels of mRNAs for both cavin1 and caveolin1 (Fig 3E).

### CD2AP co-precipitates with cavin1 and caveolin1 and regulates the sub-cellular distribution of caveolae

**Fig 4:**
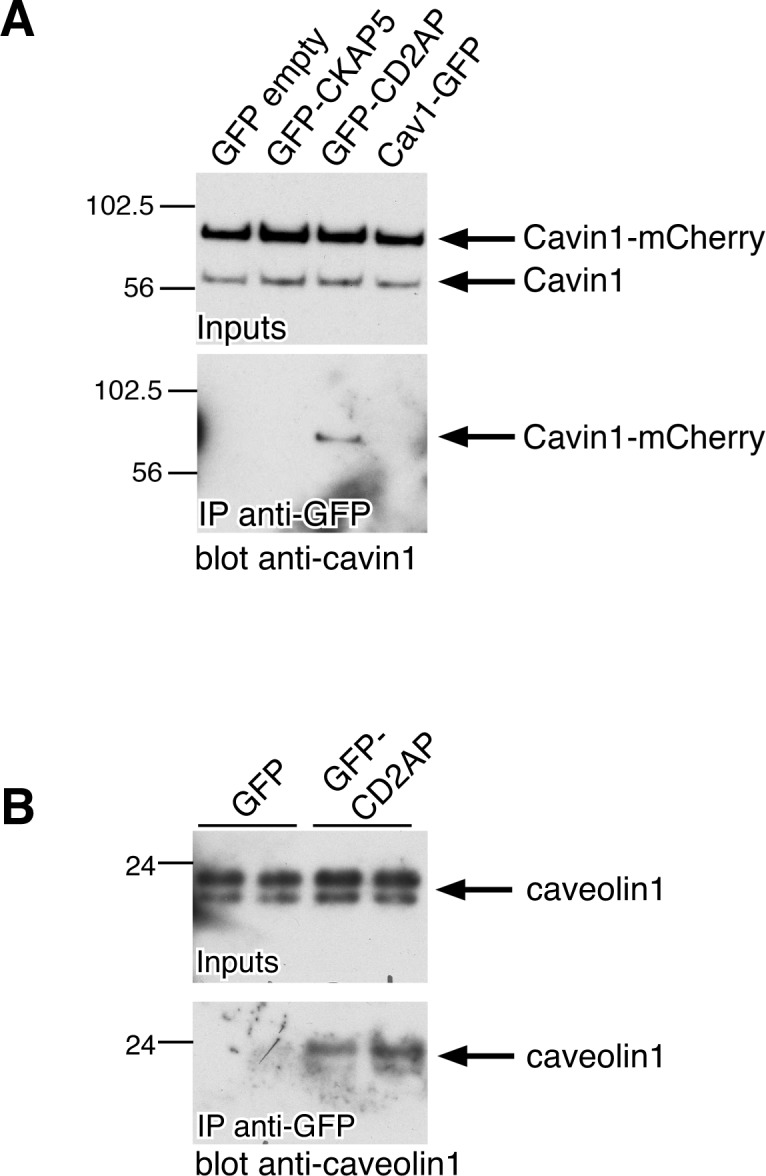
CD2AP co-precipitates specifically with cavin1 and caveolin1. **A.** Western blots labelled with anti-cavin1 antibodies to show input lysates from cells transfected with cavin1-mCherry and constructs shown for each lane, and eluates after immunoprecipitation with anti-GFP antibodies. Cells were solubilised in 0.1% TritonX100 and lysates cleared of insoluble material by centrifugation at 100,000g. Note that even low concentrations of detergent separate complexes of cavin and caveolin proteins. **B.** Western blots labelled with anti-caveolin1 antibodies to show input lysates from cells transfected with the constructs shown for each lane, and eluates after immunoprecipitation with anti-GFP antibodies. Cells were cross-linked with 0.5mM DSP prior to solubilisation in 1% octylglucoside, 1% TritonX100. Each transfection and precipitation was carried out in duplicate.

One of the potential cavin1 interaction partners, CD2AP, has previously been reported to co-precipitate with caveolin1 [38]. We expressed CD2AP as a GFP fusion in cells also expressing cavin1-mCherry. After solubilisation of caveolar proteins using Triton X100 and ultracentrifugation to remove insoluble material [19], the GFP fusion proteins were immunoprecipiated, and the presence of cavin-1-mCherry in the immunoprecipitates was assayed using anti-cavin1 antibodies. As shown in Fig 4A, GFP-CD2AP specifically co-precipitated cavin1-mCherry. Even though both proteins were overexpressed in these experiments, the data suggest that CD2AP-GFP and cavin1-mCherry can, under these conditions, enter the same protein complexes. Consistent with previous reports [19, 30], solubilisation in TX100 was sufficient to separate caveolins from cavins, as immunoprecipitation of caveolin1-GFP did not result in co-precipitation of cavin1 under these conditions (Fig 4A). We therefore carried out complimentary experiments using previously established cross-linking and solubilisation conditions that allow isolation of a large caveolar coat complex containing caveolin and cavin proteins [19], and asked whether under these conditions GFP-CD2AP co-precipitates caveolin1. Specific co-precipitation of GFP-CD2AP and caveolin1 was observed (Fig 4B). Although both the detergent-resistance of caveolin1 and the instability of cavin- and caveolin-containing complexes in detergent mean that this type of co-precipitation data must be interpreted with caution [1, 19, 28], our experiments combined with previous observations suggest that CD2AP can enter into the same complexes as components of caveolae [38]. For this reason we focussed further experiments on characterising the functional relationship between CD2AP and caveolae.

**Fig 5:**
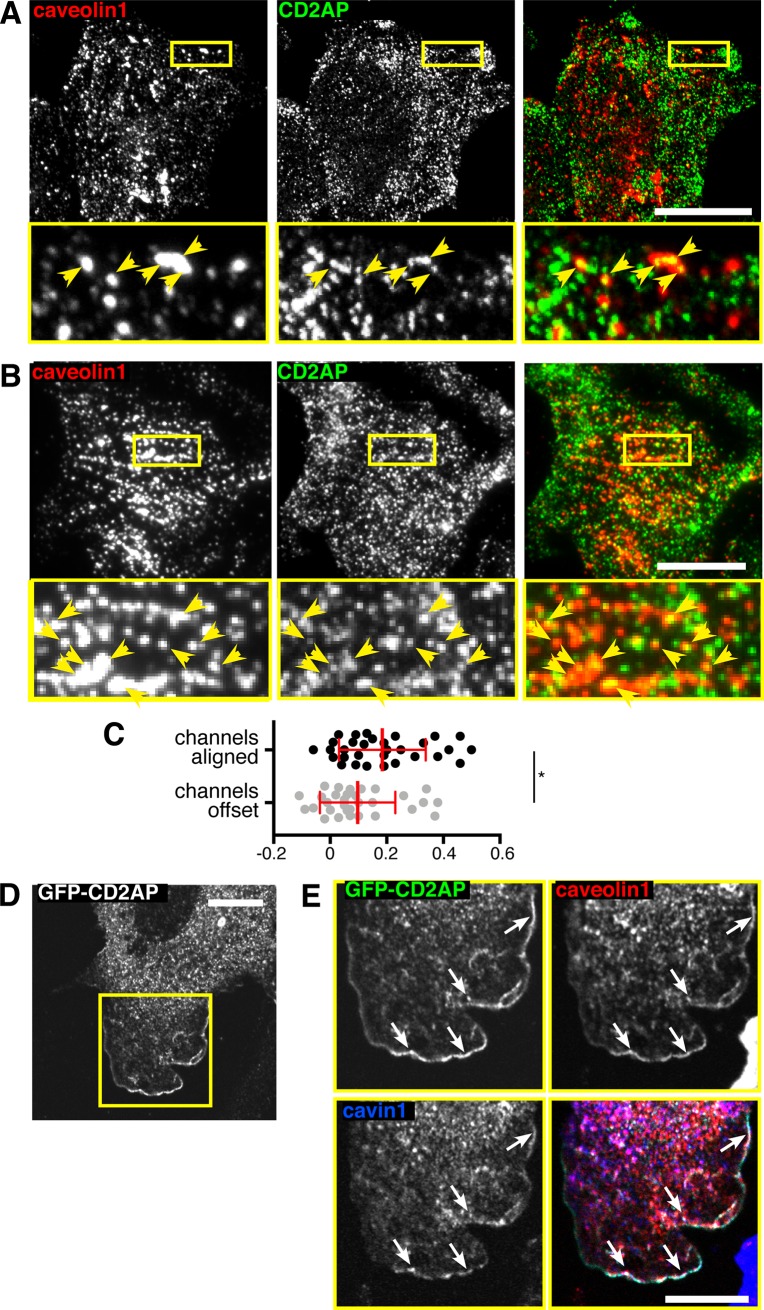
CD2AP co-localises with components of caveolae. **A**. HeLa cells labelled with anti-caveolin1 and anti-CD2AP antibodies. The region in the yellow box is shown magnified in the lower panels. Bar 20 microns. Maximum intensity projections of multiple confocal sections acquired at 1 micron intervals, with 63x objective. Yellow arrowheads indicate co-localisation. **B**. HeLa cells labelled with anti-caveolin1 and anti-CD2AP antibodies. The region in the yellow box is shown magnified in the lower panels. Bar 20 microns. Total Internal Reflection imaging, with 63x objective. Yellow arrowheads indicate co-localisation. **C**. Quantification of Pearson’s correlation coefficient in multiple cell areas from TIR images as shown in B, in either images where the two fluorescence channels are correctly aligned or where they were manually offset by approximately 0.5 microns. Statistical comparison is by t-test (* denote P<0.05). Each dot represents one cell. **D**. Cell projection induced by overexpression of GFP-CD2AP Bar 5 microns. **E**. Co-localisation between GFP-CD2AP, caveolin1 antibody labelling, and cavin1-mCherry, in the cell projection shown in D. Single confocal sections acquired with 63x objective, bar is 5 microns. White arrows indicate co-localisation.

CD2AP links the cell adhesion protein nephrin to the actin cytoskeleton, binds to actin-regulating proteins, such as CAPZ and cortactin, and specifically interacts with the C-terminal domain of Rac1 [39–47]. We began by identifying an antibody against CD2AP that provides specific labelling in indirect immunofluorescence experiments, as judged by depletion of CD2AP expression using siRNAs (S2 Fig). Indirect immunofluorescence using CD2AP and caveolin1 antibodies and confocal microscopy revealed although CD2AP was absent from the great majority of caveolin1-positive caveolae, it was clearly recruited to a small sub-population (Fig 5A). In order to gain increased resolution in the Z direction, and therefore reduce the possibility of artifactual co-localisation due to superimposition of objects in different Z locations, we supplemented confocal microscopy with Total Internal Reflection (TIR) imaging of CD2AP and Caveolin1 labelling at, or adjacent to, the plasma membrane (Fig 5B). Again, although most caveolin1-positive puncta did not have CD2AP in them, some clear co-localisation was observed. In order to quantify this, we calculated Pearson’s correlation coefficient between the caveolin1 and CD2AP channels for both images where the channels were correctly aligned, and when the channels had been offset by approximately 0.5 microns. This provides an empirical method to assess whether the detected co-localisation in specific or due to stochastic overlap of signals [26, 48]. The correlation between caveolin1 and CD2AP was, by this criterion, specific (Fig 5C).

Overexpression of GFP-CD2AP has been previously observed to induce the formation of characteristic cell protrusions (Fig 5D) [49]. In order to facilitate detection of all three markers, GFP-CD2AP was overexpressed in NIH3T3 cells genome-edited to express cavin1-mCherry from an endogenous *CAVIN1* locus [26]. Cavin1-mCherry and caveolin1 were recruited to GFP-CD2AP-induced protrusions at precisely the same peripheral location at GFP-CD2AP (Fig 5E). These observations provided additional evidence that CD2AP can enter the same complexes and caveolar components and thereby, at least in this artificial situation, influence their the sub-cellular distribution.

### CD2AP is required for recruitment of caveolae to characteristic parallel arrays adjacent to cell-cell junctions

**Fig 6:**
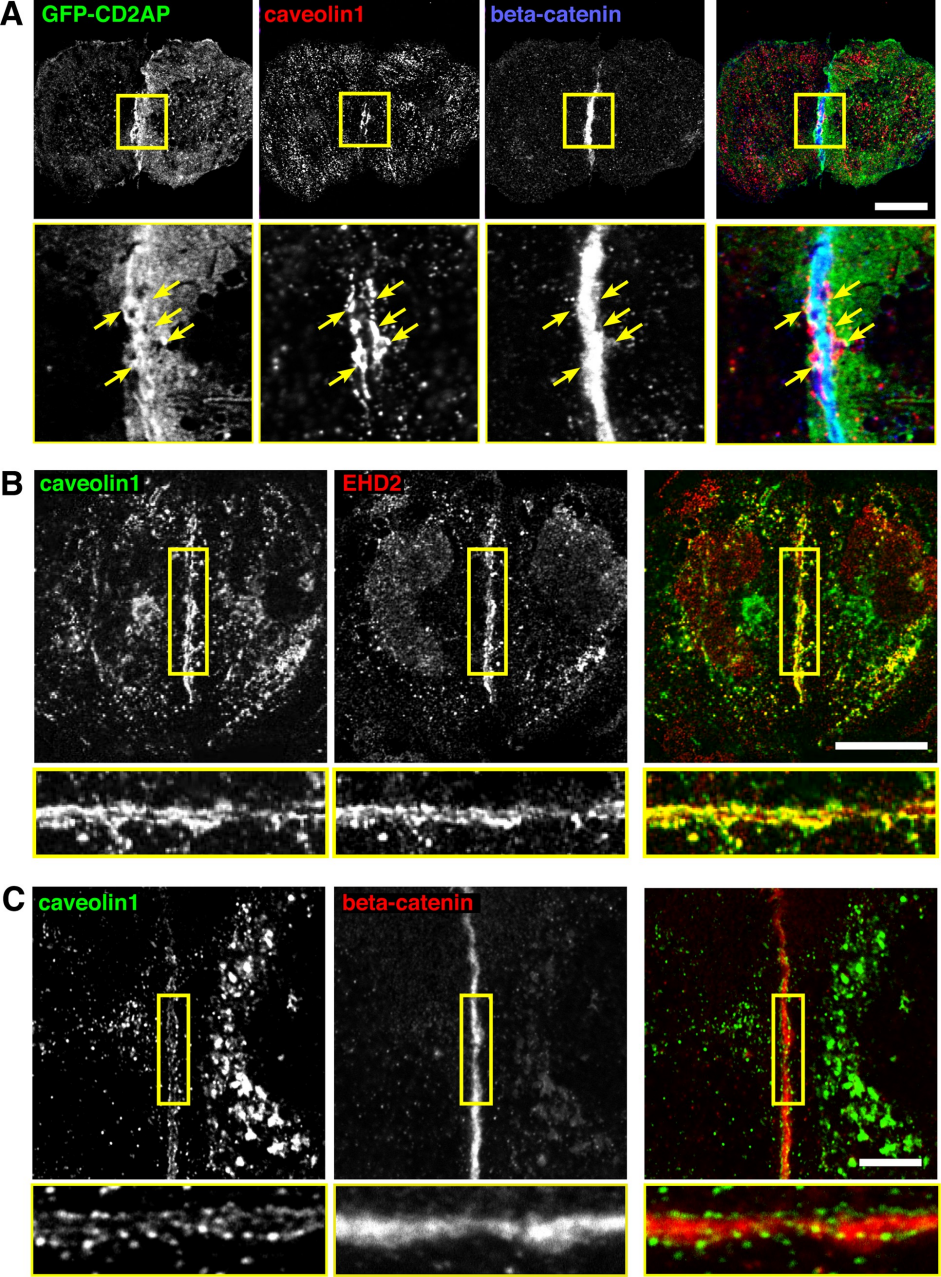
Recruitment of caveolin1 adjacent to cell junctions. **A**. HeLa cells overexpressing Rac1Q61L-myc and GFP-CD2AP, labelled with anti-caveolin1 and anti-beta-catenin antibodies. The region in the yellow box is shown magnified in the lower panels. Bar 20 microns. Maximum intensity projections of multiple confocal sections acquired at 1 micron intervals, with 63x objective. **B**. HeLa cells overexpressing Rac1Q61L-myc, labelled with anti-caveolin1 and anti-EHD2 antibodies. The region in the yellow box is shown magnified in the lower panels. Bar 20 microns. Maximum intensity projections of multiple confocal sections acquired at 1 micron intervals, with 63x objective. **C**. MDCK cells labelled with anti-caveolin1 and anti-beta-catenin antibodies. The region in the yellow box is shown magnified in the lower panels. Bar 10 microns. Single confocal section, with 63x objective.

While the observations outlined above are consistent with CD2AP entering the same protein complexes as cavin1, the functional significance of this potential interaction was not clear. Accordingly, we studied a situation where CD2AP has been shown to redistribute dynamically. In HeLa cells over-expressing the constitutively active form of Rac1, Rac1Q61L, CD2AP is recruited to cell-cell contacts where it participates in the control of epithelial barrier function [45]. We asked how caveolar components are distributed under similar conditions, as it has already been reported that the distribution of caveolin1 is regulated by the formation of cell-cell contacts [50]. In HeLa cells overexpressing Rac1Q61L beta-catenin was specifically recruited to cell-cell contacts, indicating that a functional connection between cells was formed (S3 Fig). In these cells GFP-CD2AP was also concentrated close to the beta-catenin-positive junction (Fig 6A). Caveolin1 had a distinctive, and to our knowledge previously unreported, distribution. Caveolin 1 was observed in two parallel linear arrays around 0.5 microns apart, on either side of the Rac1Q61L-induced cell-cell junction. GFP-CD2AP was also present in this junctional region, where it partially overlapped with the distribution of caveolin1 (Fig 6A). EHD2, an additional caveolar protein that does not form part of the same complex as cavins and caveolins, had the same distribution (Fig 6B).

It was important to ascertain that the distinctive parallel arrays of caveolae described above are not only found in cells overexpressing Rac1Q61L. We examined MDCK cells at early stages of polarisation, where beta catenin is prominently recruited to points of cell contact (Fig 6C). Strikingly, parallel arrays of caveolae on either side of the junction were observed in this situation also (Fig 6C). In addition, parallel arrays were not solely induced by CD2AP over-expression, as they could readily be observed in these untransfected MDCK cells (Fig 6C, S4 Fig).

**Fig 7:**
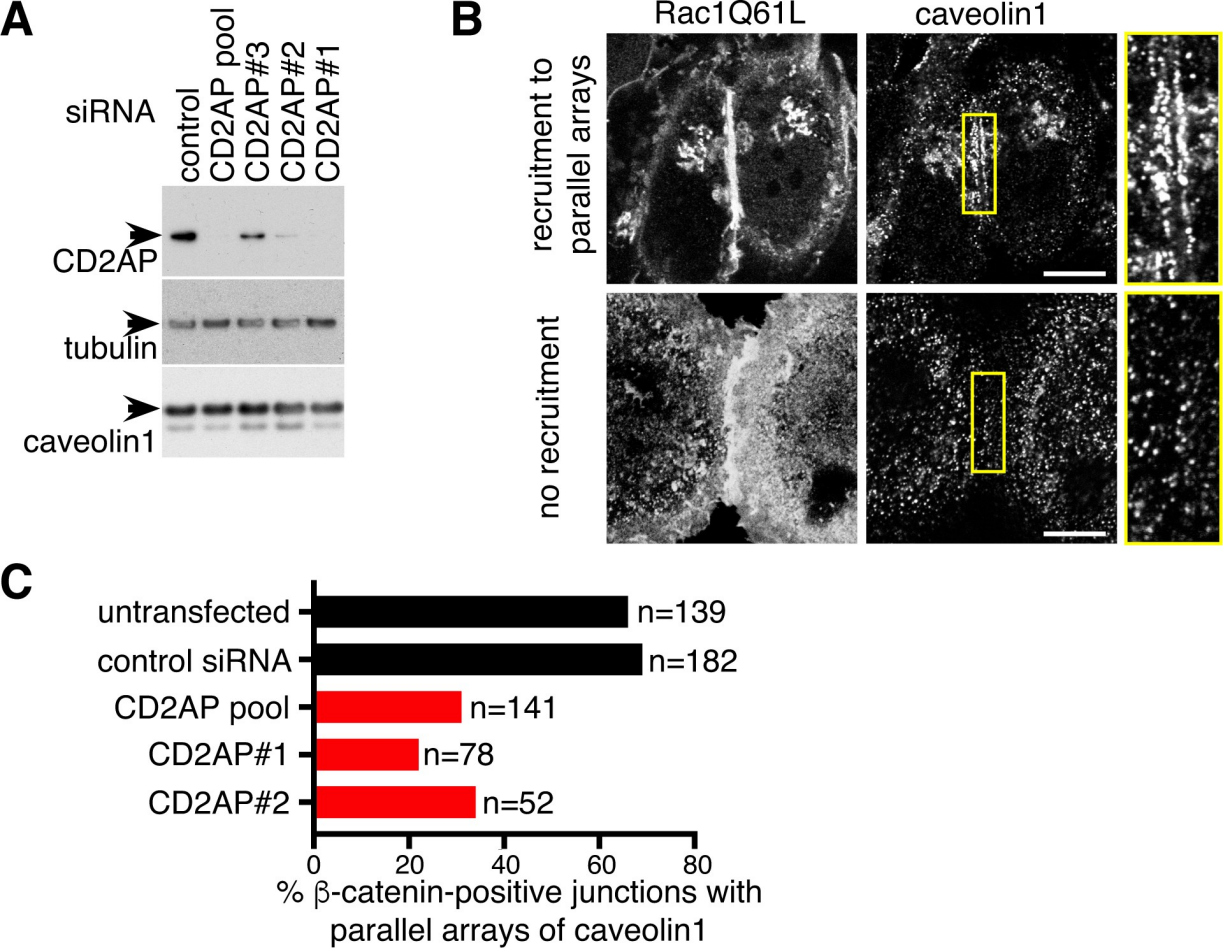
CD2AP is required for recruitment of caveolin1 to cell junctions. **A**. Western blot of cells transfected with the siRNAs shown, using antibodies as indicated. CD2AP siRNAs were either three separate single species or a pooled population containing all three. **B**. Hela cells overexpressing Rac1Q61L-myc, showing different degrees of recruitment of caveolin1 to cell-cell junctions. These categories were used in the analysis shown in C below. Bars 5 microns, single confocal sections acquired with 63x objective **C**. Analysis of the recruitment of caveolin1 to cell-cell junctions, as in B, in cells treated with the siRNAs shown stained with anti beta-catenin and anti caveolin 1 antibodies. N = total number of beta-catenin-positive cell-cell junctions analysed.

Transfection with siRNAs to reduce expression of CD2AP allowed us to ask whether CD2AP is involved in the re-localisation of caveolar components to parallel arrays at cell-cell contacts. These experiments were performed using HeLa cells overexpressing Rac1Q61L as a model, as the siRNA knockdown of CD2AP is efficient in these cells (Fig 7A). The distribution of caveolin1 at contacts defined by the recruitment of beta-catenin (Fig 6A and S3 Fig) was classified according to the presence or absence of parallel arrays (Fig 7B). CD2AP siRNAs had a profound effect on the distribution of caveolin1, reducing the recruitment to parallel arrays (Fig 7C). The recruitment of beta-catenin to cell-cell contacts was not visibly altered by the same siRNAs (S5 Fig). These findings suggest that CD2AP has an important function in controlling the sub-cellular distribution of caveolae under these conditions.

## Discussion

Several factors suggest that our list of potential interaction partners for cavin1 contains proteins that do indeed interact with cavin1 in a biologically relevant way.

First, the list contains other cavin and caveolin proteins expressed in the HeLa cells we used, and the enrichment scores for the novel candidates we have identified are all greater than that obtained for caveolin1. Caveolin proteins are difficult to detect by mass spectrometry, presumably because of their high hydrophobicity [19]. Successful identification of a set of cavin and caveolin proteins provides a strong indication that our BirA*-fusion-based approach is suitable for identifying proteins that interact with cavin1.

Second, the list contains a further protein, thioredoxin reductase, previously shown to be recruited to caveolae [34].

Third, our initial characterisation of the potential interaction partners reveals that one (CSDE1) regulates mRNA and protein levels for multiple components of caveolae, implying a functional relationship, and a second (CD2AP) controls the recruitment of caveolae to cell-cell junctions. Additionally, CD2AP can be specifically co-precipitated with cavin1 [38].

We have not detected extensive co-localisation between any of the new potential interaction partners and cavin1 (S6 Fig). It may be that the reagents we used, including commercially-produced antibodies and fluorescent protein fusions, do not correctly report the distribution of the relevant endogenous proteins. Another possibility is that the actual interactions in cells are spatially and temporally regulated so that only a minor fraction of the total amount of each protein present in cells is involved in the pertinent interaction at any one time. Our co-immunoprecipitation data for CD2AP and cavin1 reveal that the complex containing both proteins comprises less than 5% of the total amount of each protein present in cells. A third possibility, which we clearly cannot exclude, is that some of the potential interaction partners we have identified do not, in fact, interact with cavin1 or other caveolar components in cells.

CSDE1 (also called Unr) is an RNA binding protein that functions in post-transcriptional control of gene expression. It may regulate entry of mRNAs to ribosomes, stability of mRNAs, and translation-coupled mRNA degradation [35–37, 51–53]. An interaction between CSDE1 and cavin1 could conceivably, therefore, relate either to the role of cavin1 in regulating ribosomal RNA biosynthesis or the role of cavin1 at caveolae. The fact that depletion or overexpression of CSDE1 influences levels of both cavin1 and caveolin1 mRNAs favours the latter possibility, and clearly much more remains to be discovered about the molecular interactions leading to these effects.

CD2AP is an actin barbed-end capping protein [44], and acts to recruit cortactin to the cell periphery to facilitate formation of lamellipodia [49]. The absence of CD2AP causes reduced mechanical resilience of cell-cell adhesions in epithelial layers in cultured cells [44], and increases in both permeability of the endothelial blood-brain barrier and glomerular permeability in the kidney [54–56]. There is, therefore, good evidence that this protein plays an important role in maintaining cell-cell junctions of different kinds. Moreover, previous experiments also show that the sub-cellular distributions of both CD2AP and caveolin1 are regulated by Rac1 in a similar manner [57, 58], and it has already been reported that CD2AP and caveolin1 co-precipitate [38]. The literature also provide indications that caveolin1 directly or indirectly regulates the function of beta catenin and potentially further components of the machinery responsible for the regulation and integrity of cell-cell junctions [2, 59–62]. Changes in the morphology of junctions between endothelial cells, and increased endothelial permeability, have been observed in *CAV1* knockout mice, which do not have caveolae in non-muscle tissues [12, 63]. One possibility raised by our new observations in conjunction with the literature is that binding of CD2AP to caveolar components is involved in recruitment to specific locations in the cell, but further experiments will be needed to test this directly.

We suggest that recruitment of caveolae to membrane regions adjacent to cell-cell adhesions of different kinds could play an important role in buffering tension within the plasma membrane exerted by stretch forces across the cell-cell adhesion. Again, further functional experiments will be required to test this hypothesis.

## Materials and methods

### Cell culture and imaging

Hela cells were from ATCC (Cat# CCL-2), and were used at under 25 passages. NIH-3T3 cells were from ATCC (Cat# CRL-1658). NIH-3T3 caveolin1-EGFP and NIH-3T3 cavin1-mCherry knock-in cells have been described previously [26]. MDCK type IIG cells were from WJ Nelson lab, Stanford University. Cells were grown at 37°C in Dulbecco’s modified Eagle’s medium (DMEM, Invitrogen) supplemented with penicillin/streptomycin and 10% of heat inactivated fetal bovine serum for HeLa and MDCK cells and 10% calf serum for NIH3T3 cells. Cells were tested and free from mycoplasma contamination (in house service using MycoAlert mycoplasma detection kit, Lonza).

Cells were fixed with 4% paraformaldehyde at room temperature for 15 min. After four washes with PBS, the cells were blocked and permeabilised with 0.2% saponin, 10% FBS in PBS. After brief washing with PBS, the cells were incubated with appropriate primary antibody for 60 min at room temperature or overnight at 4°C. The cells were then washed four times for 5 min with and then incubated with the Alexa-secondary antibodies or fluorescent streptavidin for 1hr at room temperature.

### DNA plasmids and transfection

BioID plasmids were generated as follows: cDNAs from full length rat Cavin1, mouse Flotillin 1, rat Flotillin 2, human CD20, human CD8 and a myristoylation/palmitoylation sequence (GCGCSSHPEDDGGSGGSGGS) were cloned either with restriction digestion NheI and BamHI or by PCR and Gibson Assembly (NEB, Cat# E2611) into pcDNA3.1(+) with BirA* (R118G) followed by a GSGSGS linker and myc tag (EQKLISEEDL). To generate EGFP-CD2AP a 1914bp fragment corresponding to mouse CD2AP was cloned from mouse embryonic fibroblasts (MEF) cDNA using the next primers Fwd 5’ gccgcagcatggttgactatattgtggaatatgac and Rev 5’ gcaaagctgaagaaagctgttctgttgtct and cloned into pEGFPC1 using Gibson Assembly Master Mix according to the manufacturer’s instructions. IMAGE clone #5111213 was used to overexpress full length human CSDE1, mouse CKAP5 from IMAGE clone 8861354 was cloned into pEGFPC1 plasmid, and Addgene plasmid #12983 was used to express constitutive active Rac1 (pRK5-myc-Rac1-Q61L). Rat cavin1-mCherry is described in [64]. Plasmids were transfected in HeLa and MDCK cells using either FuGeneHD (Promega, Cat# E2311) or PEIMAX (Polyethyleneimine MW 40,000, Polysciences Cat# 24765) and NIH3T3 cells were electroporated using the Neon transfection system.

### siRNA knockdown

Silencer select siRNAs were purchased from ThermoFisher Scientific (Ambion) and their sequences are listed in S2 File. Three siRNAs for each gene were pooled and transfected at 5mM or used individually at 5mM concentration using Lipofectamine RNAiMAX (ThermoFisher Scientific, Cat # 13778030) following manufacturer’s instructions. Unless otherwise stated the pooled siRNAs were used–in some experiments single siRNAs were used, and this is indicated in the relevant Figures.

### Affinity capture of biotinylated proteins

The protocol for isolating proteins biotinylated by BirA* was adapted from the BioID method [31, 32]. Briefly, HeLa cells at 60% confluence grown in 15cm dishes were transiently transfected with FuGeneHD with 5ug of plasmid. One day after transfection, medium was removed and replaced with fresh medium containing 50 μM biotin (10 mg.ml^−1^ stock in dimethylsulfoxide), and the cells were incubated for a further 16-20hrs before lysis. For lysis, cells were thoroughly washed 6 times with PBS at room temperature, all remaining solution exhaustively removed and 1ml of lysis buffer (50 mM Tris pH 8, 300 mM NaCl, 5 mM EDTA, 1% Triton X-100, 1% octyl-glucoside and cOmplete inhibitor cocktail (Roche, Cat# 04693159001)) was added per dish. Cells were scraped immediately, vortexed briefly and incubated on ice for 30 min. After centrifugation at 13,000*g* for 15 min at 4 °C, the supernatants were mixed with 1 ml of 50 mM Tris pH 7.4. Five per cent of this sample was saved for immunoblotting, and the rest was added to 500 μl Dynabeads MyOne Streptavidin C1 beads (Invitrogen) that had been pre-washed twice in the same buffer. The beads were incubated at 4 °C overnight, washed twice in wash buffer 1 (2% SDS, cOmplete inhibitors), twice in wash buffer 2 (1% Triton X-100, 0.1% deoxycholate, 500 mM NaCl, 1 mM EDTA, 50 mM HEPES, cOmplete inhibitors, pH 7.5), once in wash buffer 3 (250 mM LiCl, 0.5% NP-40 alternative (IGEPAL), 0.5% deoxycholate, 1 mM EDTA, 10 mM Tris pH 8, cOmplete inhibitors, pH 8.4) and twice in wash buffer 4 (50 mM Tris pH 7.4, 50 mM NaCl, cOmplete inhibitors). Finally, the beads were incubated in 30 μl SDS sample buffer containing 250 mM DTT and 3 mM biotin at 98 °C for 5 min to dissociate the biotinylated proteins from the beads. A second elution was done and both elutions pooled. 40 ul was run on a gel for mass spectrometry, with the remainder reserved for immunoblotting.

### Immunoprecipitation

10 cm Petri dishes at 75% confluency were transfected with cavin1-mCherry and one of the following EGFP plasmids: EGFP-CKAP5, EGFP-CD2AP, caveolin1-EGFP or empty pEGFPN1 plasmid. Cells were thoroughly washed in PBS, lysed and scraped with lysis buffer (0.1% TritonX100, 10 mM Tris pH8, 150 mM NaCl, 0.5 mM EDTA and protease inhibitors) and incubated for 30 min on ice. Samples were centrifuged at 50.000 rpm (100,000 g) for 30 min at 4°C and the GFP-tagged proteins from the supernatant were isolated by incubation with 10 ul per sample of packed magnetic GFP-Trap agarose beds (Chromotek Cat# gtma-20) overnight at 4°C. Using magnetic stand separation the samples were washed 3 times with lysis buffer without TritonX100 and eluted with 50ul 2X SDS buffer with 150 mM DTT.

For Fig 4B, transfected cells were chemically cross-linked as previously described [19] with the only modification that the cells were cross-linked for 30min at room temperature with 0.5mM DSP. After quenching and washes, cells were scraped in 1% octylglucoside, 1% TritonX-100, 50mM Tris pH8, 300mM NaCl, 5mM EDTA and protease inhibitors. Immunoprecipitation was performed as described and washes done using the same 1% octylglucoside, 1% TritonX-100 lysis buffer.

### Quantitative PCR

Total RNA was extracted using RNeasy mini kit (Qiagen, Cat# 74104) following manufacturer instructions and DNA was removed with on-column DNaseI digestion (RNase-free DNaseI, Qiagen #79254). RNA was reverse transcribed using the High-Capacity RNA-to-cDNA Kit (Applied Biosystems, Cat# 4387406). Quantitative PCR analysis was made using delta-delta-CT method with TaqMan probes from ThermoFisher Scientific (FAM-MGB as dye-quencher) using TaqMan Universal Master Mix II, with UNG (Applied Biosystems, Cat# 4440038) on a ViiA7 Real-Time PCR System (Applied Biosystems). Hprt was used as a housekeeper gene for normalization. The following human probes were used: Cav1 (Hs00971716_m1), Cavin1 (Hs00396859_m1), Hprt (Hs99999909_m1) and CSDE1 Hs00918650_m1).

### Antibodies

The following antibodies were used: rabbit anti-caveolin1 (BD Biosciences Cat# 610060), mouse anti-βeta-catenin (BD Biosciences Cat# 610153), rabbit anti-cavin1 (Abcam Cat# ab48824), rabbit anti CD2AP (A599 Cell Signalling, Cat# 5478) for WB and mouse anti-CD2AP (Santa Cruz Biotechnology Cat# sc-25272) for immunostaining, YL1-2 anti-alpha tubulin (in-house cell culture supernatant), mouse anti-GFP (Roche Cat# 11814460001), mouse anti-flotillin-2 (BD, 610384), goat anti-EHD2 (Abcam Cat# ab23935), rabbit anti-CSDE1 (N2C1 GeneTex, Cat# GTX116218), mouse anti-TXNRD1 (Novus Biologicals Cat# NBP2-59489), mouse anti-vigilin (LSBio Cat# LS-C342610-100), rabbit anti-PRRC2C (Abcam Cat#ab117790), rat anti-Nav1 (Abcam Cat#ab201920) and mouse anti-myc clone 9E10 (in-house cell culture supernatant). Horseradish peroxidase (HRP)-conjugated secondary antibodies were from DAKO and Streptavidin HRP from Cell Signalling (Cat# 3999). Streptavidin Alexa 647 and Alexa Fluor fluorescent secondary antibodies were from Life Technologies (Thermo Fisher).

### Microscopy

All confocal imaging was carried out using a Zeiss LSM710 inverted confocal microscope with a 63x/1.4NA oil objective, driven by Zen software. TIR imaging was carried out using a specifalised 63x/1.4NA TIR objective on an Olympus CellView system.

### Mass spectrometry

Samples were loaded on 4–12% Bis-Tris SDS–PAGE gels and run for 4–5 centimetres. Proteins were stained with Coomassie InstantBlue (Expedeon), the entire gel lane was cut into eight slices that were placed in a 96-well plate and destained with 50% v/v acetonitrile and 50 mM ammonium bicarbonate, reduced with 10 mM DTT, and alkylated with 55 mM iodoacetamide. Digestion was with 6 ng/μl trypsin (Promega, UK) overnight at 37°C, and peptides extracted in 2% v/v formic acid 2% v/v acetonitrile, and analysed by nano-scale capillary LC-MS/MS (Ultimate U3000 HPLC, Thermo Scientific Dionex) at a flow of ~ 300 nL/min. A C18 Acclaim PepMap100 5 μm, 100 μm x 20 mm nanoViper (Thermo Scientific Dionex), trapped the peptides prior to separation on a C18 Acclaim PepMap100 3 μm, 75 μm x 250 mm nanoViper. Peptides were eluted with an acetonitrile gradient. The analytical column outlet was interfaced via a nano-flow electrospray ionisation source with a linear ion trap mass spectrometer (Orbitrap Velos, Thermo Scientific). Data dependent analysis was performed using a resolution of 30,000 for the full MS spectrum, followed by ten MS/MS spectra in the linear ion trap. MS spectra were collected over a m/z range of 300–2000. MS/MS scans were collected using a threshold energy of 35 for collision-induced dissociation. LC-MS/MS data were searched against the UniProt KB database using Mascot (Matrix Science), with a precursor tolerance of 10 ppm and a fragment ion mass tolerance of 0.8 Da. Two missed enzyme cleavages and variable modifications for oxidised methionine, carbamidomethyl cysteine, pyroglutamic acid, phosphorylated serine, threonine and tyrosine were included. MS/MS data were validated using the Scaffold programme (Proteome Software Inc).

### Quantification, statistical analysis

Enrichment scores for each protein in each individual BioID experiment were calculated as follows. The number of unique peptides from that protein identified in the cavin1-BirA* sample (exclusive peptide count) was divided by 1 + the sum of exclusive peptide counts for that protein in all of the negative control samples for that experiment. The negative controls applied were not the same in all of the BioID experiments, their identity is detailed in S1 File, which also includes mass spectrometry data for each experiment. The ‘normalised product of scores’ was generated by multiplying together (enrichment score + 1) for each experiment, and then normalising to the result in the case of caveolin1.

The quantification of the effect of CD2AP knockdown on Cav1 recruitment to cell-cell contacts in Rac1Q61L transfected cells (Fig 7C) was done as follows: 15 confocal images for each condition (control without siRNA, siRNA negative control and siRNA CD2AP) in different slide areas were acquired using 63X lens and no optical zoom. Then, the number of cell-cell contacts positive for beta-catenin was manually counted and that corresponded to the total of contacts. From those beta-catenin-positive contacts, caveolin1 recruitment was manually quantified according to the presence or absence of parallel arrays of caveolae as shown in Fig 7B.

Co-localisation between CD2AP and caveolin1 (Fig 5C) was quantified using the Coloc2 plugin implemented in ImageJ to calculate Pearson’s correlation coefficient between the two fluorescence channels in manually defined cell areas. Channels were offset by approximately 0.5 microns by hand, and then the same cell areas were used for the control analysis.

### Western Blots

Samples were lysed in 1X sample buffer (NuPage LDS Sample buffer, Invitrogen Cat# NP0007) supplemented with 150mM DTT, boiled and run on precasted 4–12% Bis-Tris gels (Invitrogen) in either 1X MOPS or 1X MES buffer. The gels were then transfer into PVDF membranes and the membranes blocked in a PBS solution containing 5% dried skimmed milk powder, incubated with the appropriate primary antibodies, washed 0.1% Tween-20/PBS solution and incubated with HRP conjugated secondary antibodies (DAKO). After washing, the blots were then developed using Immobilon Western Chemiluminescent HRP Substrate (Millipore) or ECL Western Blot Detection Reagent Kit GE Healthcare Cat# RPN2209 onto Fuji Super RX X-ray films.

## Supporting information

S1 Fig Effect of siRNAs against potential caveolae-interacting proteins on caveolin1 expression. Western blots with the antibodies shown, in cells transfected with pooled siRNAs as indicated. Approximate positions of molecular weight markers are indicated (kDa), and the predicted molecular weight of each candidate is indicated in parentheses.
(TIF)

S2 Fig SiRNAs against CD2AP demonstrate that indirect immunofluorescence labelling with anti-CD2AP antibodies results in a specific signal. HeLa cells transfected with siRNAs as shown fixed and stained with anti-CD2AP antibody.
(TIF)

S3 Fig Overexpression of Rac1Q61L induces recruitment of beta-catenin to cell-cell contacts in HeLa cells. **A**. Indirect immunofluorescence with anti-myc and anti-beta-catenin antibodies. **B**. Indirect immunofluorescence with anti-beta-catenin antibodies, comparing fields of Rac1Q61L-transfected and untransfected cells.)
(TIF)

S4 Fig Caveolin1 staining in parallel arrays at junctions between polarising MDCK cells. Indirect immuniflourescence with anti-caveolin1 antibodies. The zoomed-in regions are highlighted by yellow boxes in the larger image. Bar 20 microns.
(TIF)

S5 Fig CD2AP siRNAs to not perturb recruitment of beta-catenin to cell-cell contacts. Indirect immunofluorescence with anti-caveolin1 and anti-beta-catenin antibodies in control cells and siRNA CD2AP treated cells. Cells overexpress Rac1Q61L-myc.
(TIF)

S6 Fig Imaging of potential cavin1-interacting proteins and components of caveolae. All images are single confocal sections. Unless otherwise indicates images are of indirect immunofluoresnce staining using antibodies detailed in the Methods section. NAV1 was detected using transient transfection with a GFP-Nav1 construct. Note that the distributions of MAP4 and PRRC2C were not analysed. Bars are 20 microns.
(TIF)

S1 File Mass spectrometry data from BioID experiments. Number of exclusive peptides for each protein is shown in the table. The identity of the BirA* fusion used in each sample is given at the top of each column. ‘Control’ indicates a sample where no BirA* fusion was transfected. Some experiments (F and G) were carried out in duplicate, and the values used in Fig 2 are simply the mean of the duplicates.
(XLSX)

S2 File Sequences of siRNA oligonucleotides. As shown.
(XLSX)
