# Supplementary figures and images for: BioID identifies proteins involved in the cell biology of caveolae
Source: PLoS One. 2018 Dec 27;13(12):e0209856. doi: 10.1371/journal.pone.0209856 (PMC6307745; doi:10.1371/journal.pone.0209856)

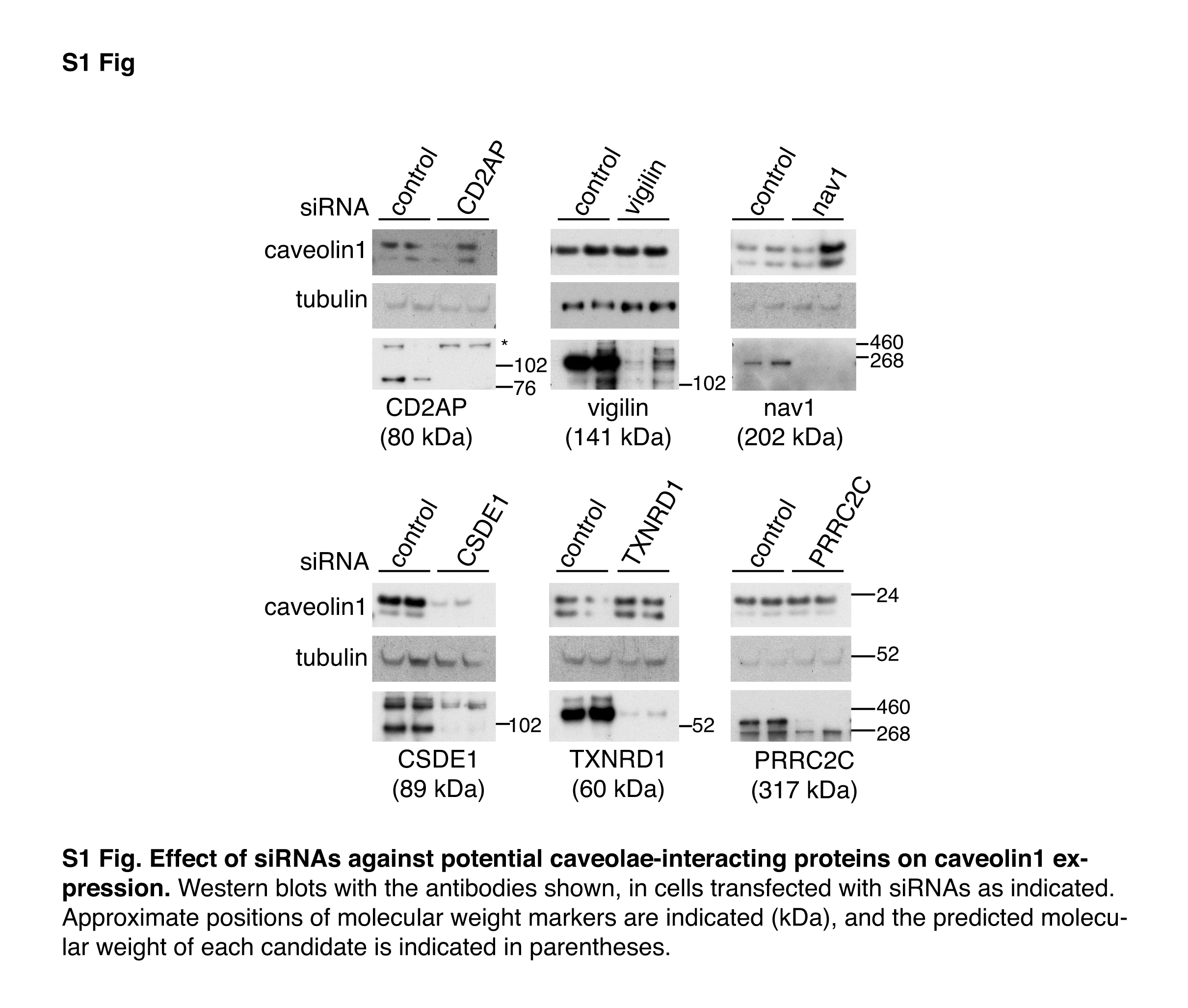

Supplement: S1 Fig — Western blots with the antibodies shown, in cells transfected with pooled siRNAs as indicated. Approximate positions of molecular weight markers are indicated (kDa), and the predicted molecular weight of each candidate is indicated in parentheses. (TIF) [file pone.0209856.s001.tif]

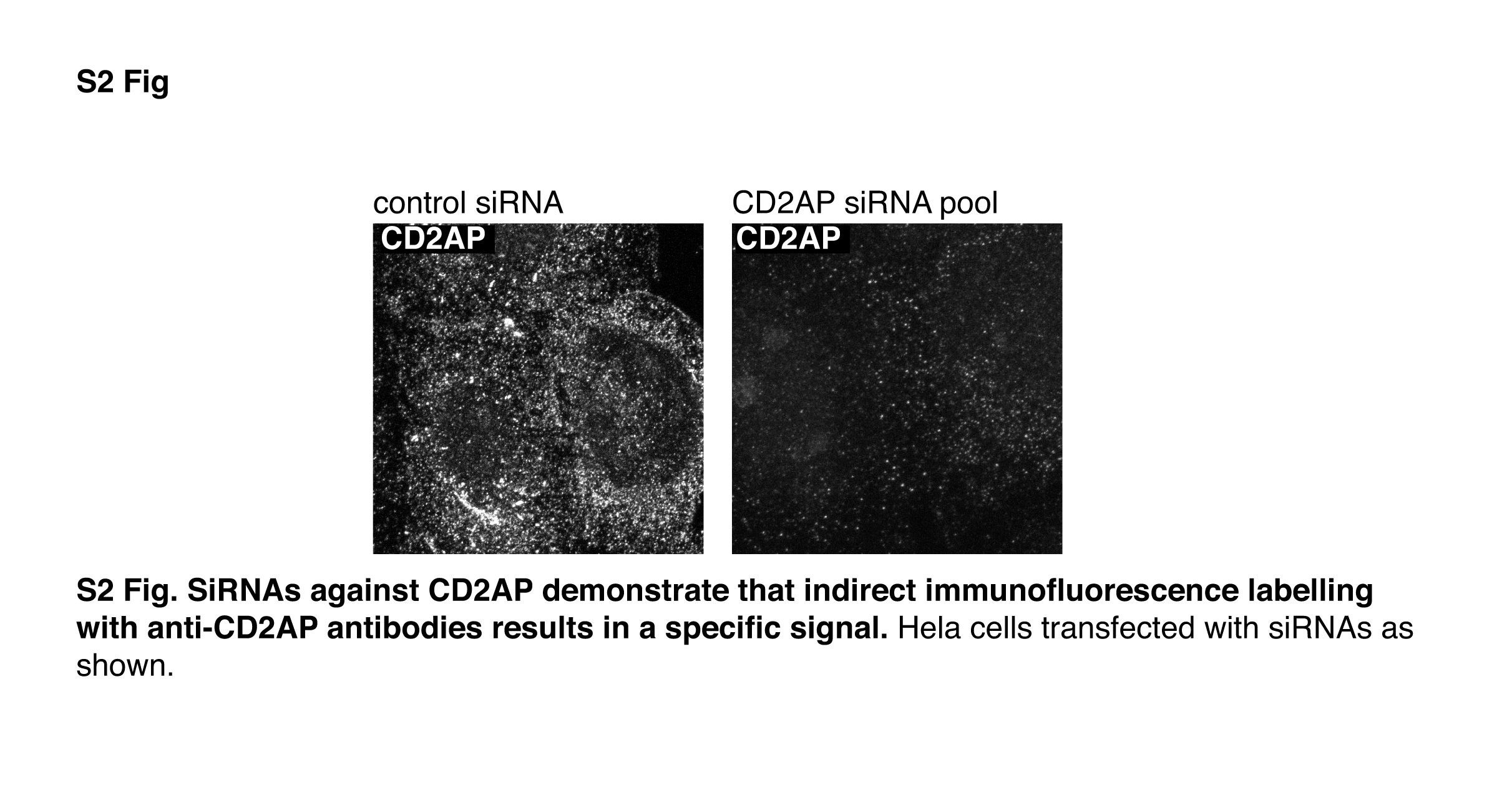

Supplement: S2 Fig — HeLa cells transfected with siRNAs as shown fixed and stained with anti-CD2AP antibody. (TIF) [file pone.0209856.s002.tif]

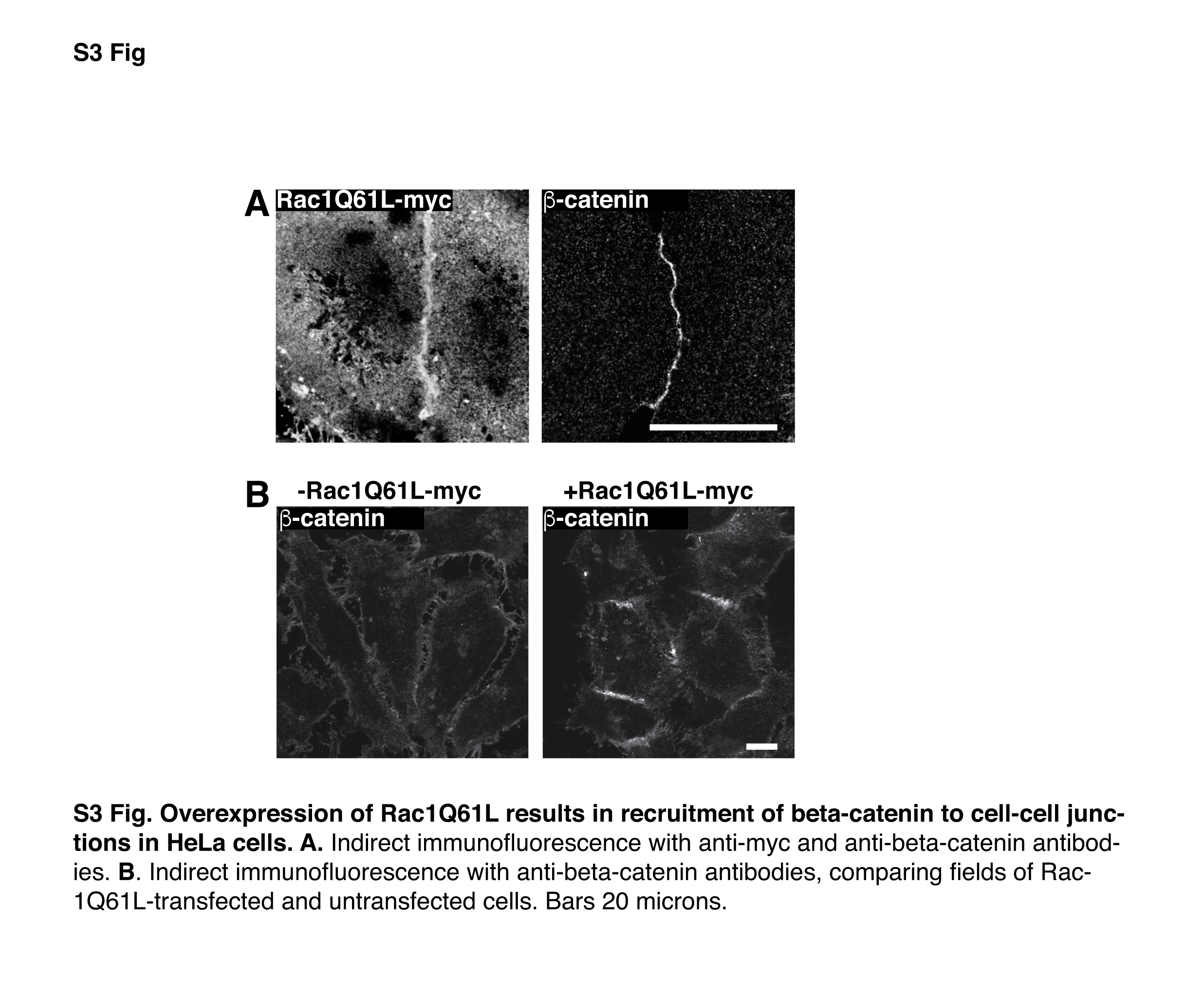

Supplement: S3 Fig — A. Indirect immunofluorescence with anti-myc and anti-beta-catenin antibodies. B. Indirect immunofluorescence with anti-beta-catenin antibodies, comparing fields of Rac1Q61L-transfected and untransfected cells.) (TIF) [file pone.0209856.s003.tif]

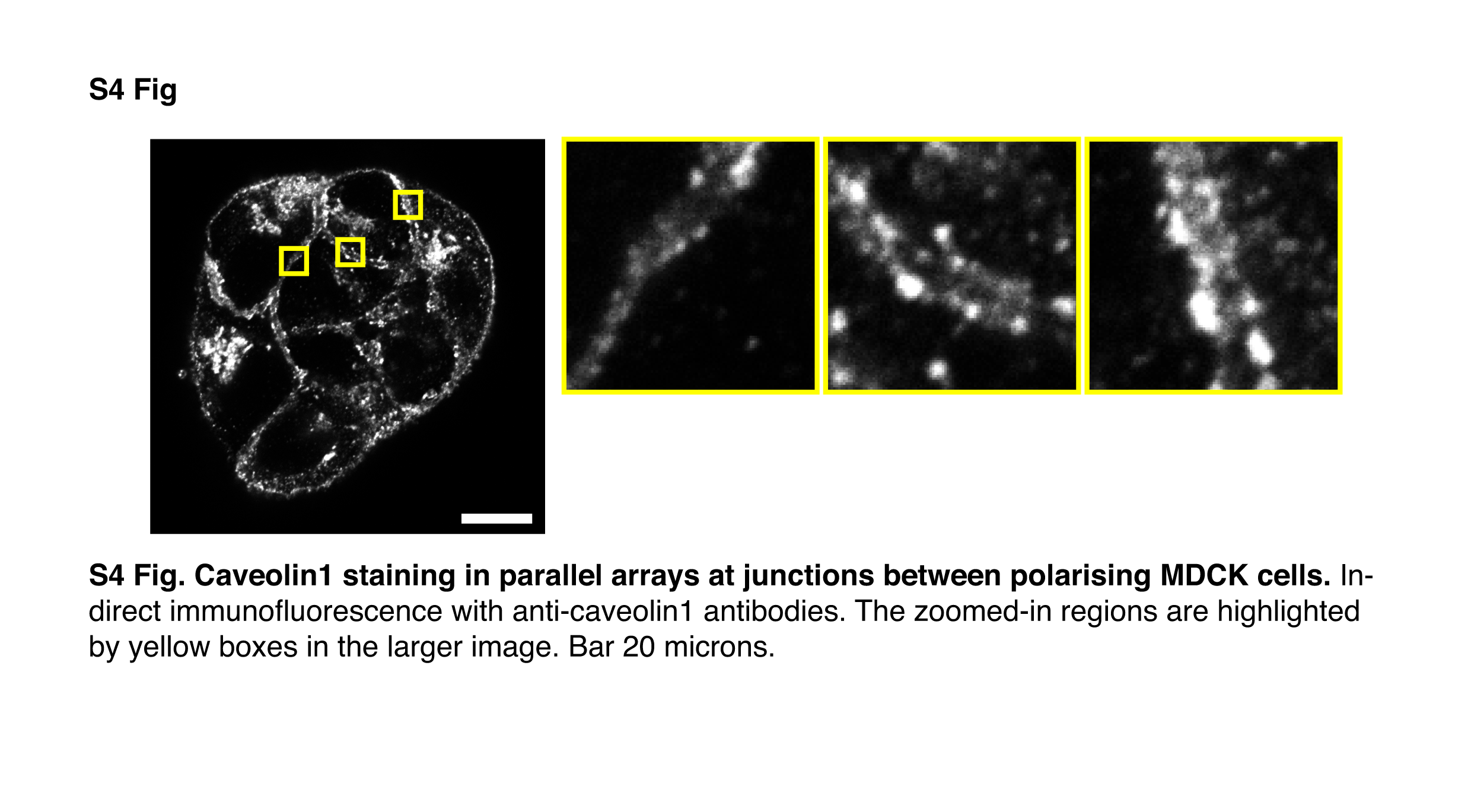

Supplement: S4 Fig — Bar 20 microns. (TIF) [file pone.0209856.s004.tif]

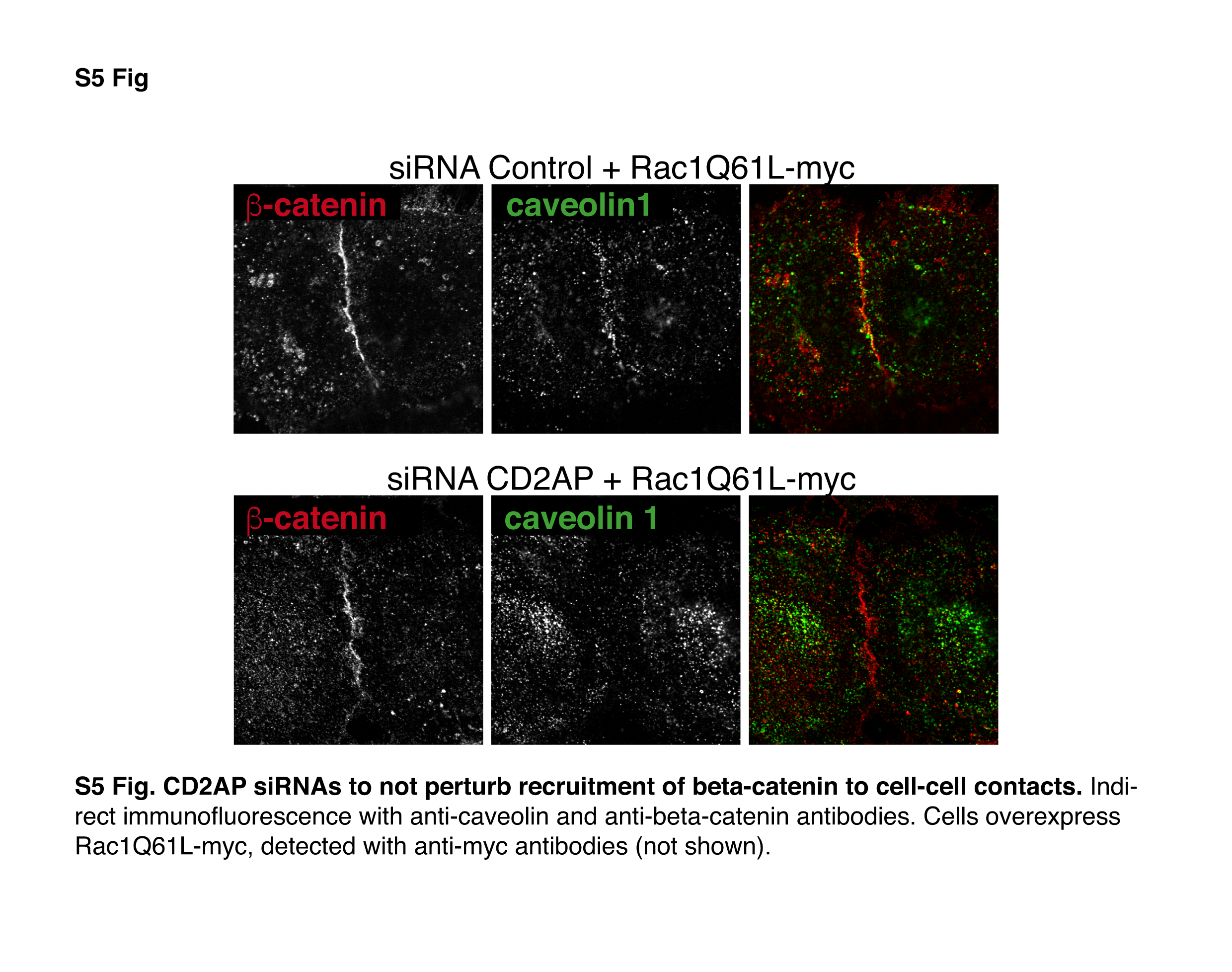

Supplement: S5 Fig — Indirect immunofluorescence with anti-caveolin1 and anti-beta-catenin antibodies in control cells and siRNA CD2AP treated cells. Cells overexpress Rac1Q61L-myc. (TIF) [file pone.0209856.s005.tif]

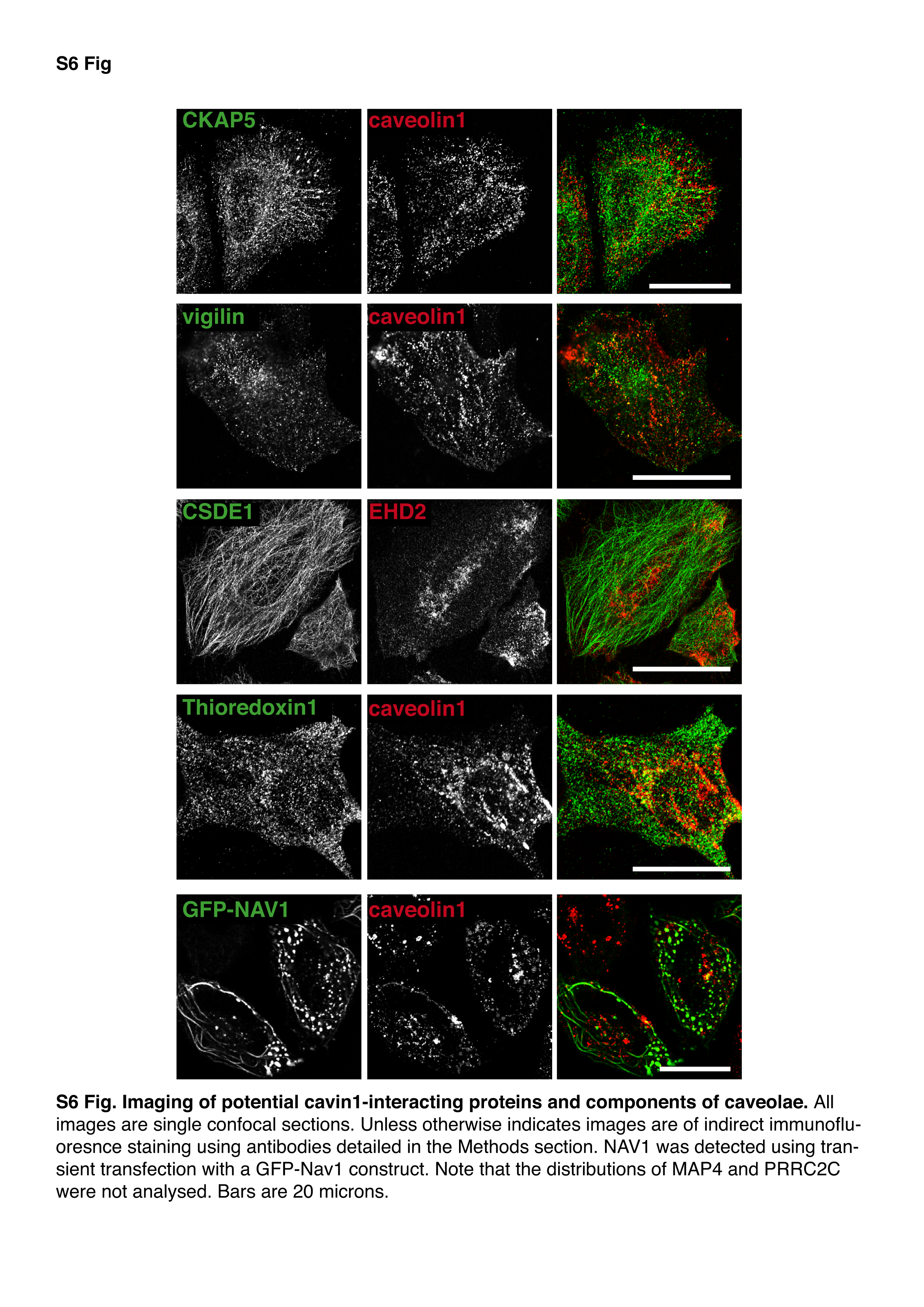

Supplement: S6 Fig — All images are single confocal sections. Unless otherwise indicates images are of indirect immunofluoresnce staining using antibodies detailed in the Methods section. NAV1 was detected using transient transfection with a GFP-Nav1 construct. Note that the distributions of MAP4 and PRRC2C were not analysed. Bars are 20 microns. (TIF) [file pone.0209856.s006.tif]
